# The Medical and Surgical Reporter

**Published:** 1858-05

**Authors:** 


THE MEDICAL AND SURGICAL REPORTER.—We learn that this journal, now well known to the profession, will hereafter be issued at Philadelphia, and that its talented editor has associated with him Dr. Atkinson, who has for some time been a contributor to its pages.

